# Self-Organization of Spatio-Temporal Hierarchy via Learning of Dynamic Visual Image Patterns on Action Sequences

**DOI:** 10.1371/journal.pone.0131214

**Published:** 2015-07-06

**Authors:** Minju Jung, Jungsik Hwang, Jun Tani

**Affiliations:** Department of Electrical Engineering, KAIST, Daejeon, Republic of Korea; Zhejiang Key Laborotory for Research in Assesment of Cognitive Impairments, CHINA

## Abstract

It is well known that the visual cortex efficiently processes high-dimensional spatial information by using a hierarchical structure. Recently, computational models that were inspired by the spatial hierarchy of the visual cortex have shown remarkable performance in image recognition. Up to now, however, most biological and computational modeling studies have mainly focused on the spatial domain and do not discuss temporal domain processing of the visual cortex. Several studies on the visual cortex and other brain areas associated with motor control support that the brain also uses its hierarchical structure as a processing mechanism for temporal information. Based on the success of previous computational models using spatial hierarchy and temporal hierarchy observed in the brain, the current report introduces a novel neural network model for the recognition of dynamic visual image patterns based solely on the learning of exemplars. This model is characterized by the application of both spatial and temporal constraints on local neural activities, resulting in the self-organization of a spatio-temporal hierarchy necessary for the recognition of complex dynamic visual image patterns. The evaluation with the Weizmann dataset in recognition of a set of prototypical human movement patterns showed that the proposed model is significantly robust in recognizing dynamically occluded visual patterns compared to other baseline models. Furthermore, an evaluation test for the recognition of concatenated sequences of those prototypical movement patterns indicated that the model is endowed with a remarkable capability for the contextual recognition of long-range dynamic visual image patterns.

## Introduction

Using a hierarchical structure has two main advantages in terms of computational efficiency and generalization mentioned by Kruger et al. [1]. By sharing the functionalities of lower levels, a hierarchical structure drastically reduces the computational cost compared to a flat structure which processes all tasks independently. At the same time, the shared functionalities of the lower levels can be generalized because these are participating in many different tasks. The brain also utilizes a hierarchy structure, especially in the visual cortex [1, 2]. The visual cortex consists of two main pathways: (1) a ventral, or what, pathway for object recognition and (2) a dorsal, or where, pathway for information processing related to position and movement [3, 4]. Both organizations of the ventral and dorsal pathway show a hierarchical structure of progressively increasing selectivity, invariance, and receptive field sizes [5].

Due to the aforementioned benefits and biological plausibility of a hierarchical structure, a machine vision scheme, the so called deep learning, has been introduced to extract high-level abstractions through multiple non-linear transformations imposed on the hierarchy [6–10]. The core idea of deep-learning-based machine vision is that all necessary information processing structures for recognizing visual image patterns are self-organized in hierarchical neural network models through iterative learning of exemplar visual image patterns. Among such models, a convolutional neural network (CNN) [6], developed using inspiration from the mammalian visual cortex for its spatial hierarchical processing of visual features, has shown remarkably superior recognition performance for static natural visual images compared to conventional vision recognition schemes which used elaborately hand-coded visual features [10]. However, in practical applications, these approaches are limited to the recognition of static visual image patterns. In other words, they cannot recognize dynamic visual image patterns extended in time because they do not comprise of any temporal processing mechanism. To overcome this limitation, it has been proposed that dynamic visual image patterns can be recognized by simply transforming a sequence of 2-dimensional visual spatial patterns within a fixed temporal window into a large 3-dimensional pattern [11–13], such as a 3D convolutional neural network (3D CNN) [11]. Indeed, these models performed well on many challenging video recognition datasets by simply extracting short-range temporal correlations. However, it cannot be extended to contextual recognition of dynamic visual image patterns which requires the extraction of relatively long-range temporal correlations. Baccouche et al. [14] has proposed a two-stage model to maintain temporal information in the entire sequence by adding a long short-term memory (LSTM) network [15] as a second stage of the 3D CNN. However, the spatial information process and the temporal one are not fully reconciled with a single principle in their model.

The current study introduces a method that can solve the problem of contextual recognition of dynamic visual image patterns by using the capability of a newly proposed neuro-dynamic model in self-organizing an adequate spatio-temporal hierarchy through only iterative learning of exemplars. The proposed model, termed a multiple spatio-temporal scales neural network (MSTNN), was constructed from two essential ideas adopted from two different existing neural network models. One is the self-organization of a spatial hierarchy through the learning of static visual image patterns seen in the CNN [6], and the other is the self-organization of a temporal hierarchy through the learning of dynamic patterns such as sensory-motor sequences seen in a multiple timescales recurrent neural network (MTRNN) [16]. The MSTNN consists of several layers, and each layer is characterized by spatial constraints: local receptive field and weight sharing of the CNN, and a temporal constraint: time constant of a leaky integrator model, which was adapted for the MTRNN. The size of the receptive fields and the timescale of dynamics gradually increase along the hierarchy. The spatial hierarchy of the receptive fields for the MSTNN is analogous to the organization observed in the mammalian visual cortex [17], and there is evidence for the assumed temporal hierarchy of progressively slower timescale dynamics of the MSTNN in the human visual cortex [18]. The premise for this assumption is that functional hierarchy could be self-organized by using both spatial and temporal constraints incorporated in the learning processes of massively high-dimensional spatio-temporal patterns present in dynamic visual image patterns.

To evaluate the performance of the MSTNN, we conducted two classes for human action recognition-by-learning tasks. Human action recognition tasks should involve sophisticated mechanisms for realizing both compositional and contextual information processing in dealing with massively high dimensional spatio-temporal patterns. The first experiment was conducted to evaluate the performance of the MSTNN in the recognition of a set of prototypical human movement patterns found in the Weizmann dataset [19] without and with occlusion by using moving stripes. In the non-occlusion case, the MSTNN had a performance comparable to two baseline models: the CNN and the 3D CNN. In the occlusion case, the MSTNN clearly outperformed the two baseline models significantly when the ratio of the occlusion was increased. The second experiment was performed to evaluate the capability of the MSTNN to recognize long-range visual images of combinatorial action sequences. The prototypical actions in the Weizmann dataset were concatenated to generate the learning and testing sequences for this experiment. The experimental results indicated that the MSTNN also performed well in this recognition task. Overall analysis of these experiments indicates that the spatio-temporal hierarchy self-organized in the MSTNN has an essential role for robust and contextual recognition of dynamic visual image patterns.

## Materials and Methods

The newly proposed model, the multiple spatio-temporal scales neural network (MSTNN), consists of multilayers of retinotopically organized neurons like the CNN. However, the MSTNN uses a leaky integrator neural unit rather than a simple static neural unit to represent spatio-temporal features at each retinotopic position at each level. The core hypothesis driving the model is that the spatio-temporal hierarchy required for contextual recognition of dynamic visual image patterns can be self-organized by imposing multiple scales of spatial and temporal constraints on the neural activity during the supervised training of a set of exemplars. Neural activation dynamics at each layer are imposed with not only a specific spatial constraint in terms of input connectivity but also a temporal constraint in terms of a time constant set for leaky integrator neural units, which are allocated in the convolutional layers. The network consisted of several layers including the raw image input layer on the bottom and the category output layer on the top. The lower layers consisted of faster dynamic neural units characterized by smaller time constants with narrower receptive fields while the higher layers consisted of slower dynamic neural units characterized by larger time constants with wider receptive fields. To reduce the enormous number of parameters of the network and tendencies for over-fitting, synaptic connections used the weight sharing scheme in which the same synaptic input weights to a feature neural unit are replicated with a shift in the entire visual field assuming spatial stationary datasets [6]. Additionally, max-pooling layers are implemented after the convolutional layers to gain spatial invariance properties [20]. The top layer consisted of a set of winner-take-all neural units representing the categorical outputs as the recognition results for the dynamic visual input. A conventional back-propagation through time algorithm (BPTT) is used for the learning processes [21]. Precise mathematical descriptions and methods for training are provided in the following sections.

### Convolutional Layer with the Leaky Integrator Model

We used a leaky integrator model, by which each neuron’s activity at each convolutional layer is calculated not only by convoluting its kernels with the corresponding feature maps in the previous layer, but also by adding its decayed internal state from the previous time step. The internal states and activation values of the *m*th feature map in the *l*th convolutional layer at time step *t*, denoted as $u_{t}^{lm}$ and $v_{t}^{lm}$, respectively, are calculated by

$$u_{t}^{lm}=(1-\frac{1}{\tau^{l}})\cdot u_{t-1}^{lm}+\frac{1}{\tau^{l}}\cdot (\sum_{n}k^{lmn}*v_{t}^{(l-1)n}+b^{lm})$$ (1)

$$v_{t}^{lm}=1.7159\cdot \tanh(\frac{2}{3}\cdot u_{t}^{lm})$$ (2)

where * is a convolution operation, *τ^l^* is the time constant of the *l*th layer, **k** ^*lmn*^ is the kernel weights connected from the *n*th feature map in the (*l*−1)th layer to the *m*th feature map in the *l*th layer, and *b^lm^* is the bias for the *m*th feature map in the *l*th layer. As represented by the first RHS term of Eq (1), the decay rate of previous internal states **u**_*t*-1_ depends on the time constant *τ*. In other words, the time constant *τ* represents the timescale of dynamics or implicit size of the temporal window at each layer. A smaller and a larger time constant result in faster and slower timescale dynamics, respectively. By virtue of the characteristics of the leaky integrator neuron model, MSTNN can comprise the temporal hierarchy with few additional time constant parameters by simply assigning smaller time constants to lower layers and larger time constants to higher layers. For the activation function of the convolutional layer, we use a scaled version of the hyperbolic tangent function recommended by LeCun et al. [22].

### Delay Response Method

Conventional temporal classifiers generate output results and errors at each time step. However, in this paper, the classification and training are done in a delay response manner, hereafter called a delay response method. In the delay response method, the target categorical outputs are presented and compared with the predicted categorical outputs only after each visual stream input is terminated. By passing very deep non-linear functions in terms of space and time through the network, the MSTNN can utilize complete and refined spatio-temporal information at the end of dynamic visual image patterns.

The error function *E* is determined using the Kullback-Leibler divergence defined as follows:

$$E=\sum_{t=1}^{T}E_{t}$$ (3)

$$E_{t}=\{\begin{array}{cc} \sum_{c=1}^{C}y_{c}^{*}\ln(\frac{y_{c}^{*}}{y_{tc}}) & \text{if}\;T-t\leq d\\ 0 & \text{otherwise} \end{array}$$ (4)

where *T* is the length of the visual sequence containing (1) the original visual stream and (2) black frames which are added at the end of the original visual stream to generate output results and errors in the delay response method, *d* is the length of the black frames which is equal to that of the label sequence, *C* is the total number of classes within a dataset, *y_tc_* represents confidence in class *c* when provided with the visual sequence at time step *t*, and y* is the true class distribution. If an input visual sequence belongs to class *c*, $y_{c}^{*}$ is set to 1 while the rest of the entries of y* are set to 0. Because the model is trained in the delay response method, error *E_t_* at each time step is only generated in the final *d* time steps of the visual sequence.

The predicted probability distribution **y**_*t*_ over all classes *C* at time step *t* are generated by taking the softmax function over the internal states **s**_*t*_ in the output layer of the MSTNN in response to the visual sequence at time step *t*:

$$y_{tc}=\frac{\exp(s_{tc})}{\sum_{c^{\prime }=1}^{C}\exp(s_{tc^{\prime }})}$$ (5)

### Details of Training

The learnable parameters of the network, here the kernel weights **k** and biases **b**, are denoted by ***θ***. We implement a stochastic gradient descent method during the training phase, where the learnable parameters of the network ***θ*** are updated to minimize the error function defined in Eq (3) after each visual sequence is given. Additionally, a weight decay of 0.0005 is applied while updating the kernel weights to prevent over-fitting as used by Krizhevsky et al. [10]. The update rules for the learnable parameters ***θ*** are expressed as follows:

$$k\;(n)=k\;(n-1)-\alpha \cdot (\frac{\partial E}{\partial k}+0.0005\cdot k\;(n-1))$$ (6)

$$b\;(n)=b\;(n-1)-\alpha \cdot \frac{\partial E}{\partial b}$$ (7)

where *n* is the epoch index, *α* is the learning rate, and $\frac{\partial E}{\partial \theta }$ is the partial derivative for each learnable parameter. $\frac{\partial E}{\partial \theta }$ is normalized by dividing the length of the given visual sequence *T* before using it for updating the learnable parameters. $\frac{\partial E}{\partial \theta }$ can be solved using a conventional back-propagation through time method (BPTT) [21].

All kernel weights and biases were initialized using randomly selected values from a zero-mean Gaussian distribution with standard deviation of 0.05. The initial internal state for each of the neurons in all convolutional layers was set to 0. The length of the black frames was set to 15. The learning rate was initialized to 0.1. To accelerate training, we used an adaptive learning rate method. If the mean square error (MSE) calculated subsequent to one epoch is smaller than the previous one, then the learning rate is multiplied by 1.05; otherwise, the learning rate is divided by 2.

### Overall Architecture

There are two generally accepted guidelines to set the parameters of the CNN. The first guideline is that the feature map size or the resolution of the feature map is decreased with depth to efficiently compress the number of dimensions within a massively high dimensional visual input. The second guideline is that the number of feature maps, which determines how many features are extracted in each layer, is increased with depth to compensate for the loss of spatial information because of the first guideline. We followed these two guidelines and an additional guideline that the time constant increases along the hierarchy to set the parameters of the MSTNN which are used in the described experiments.

**Fig 1:**
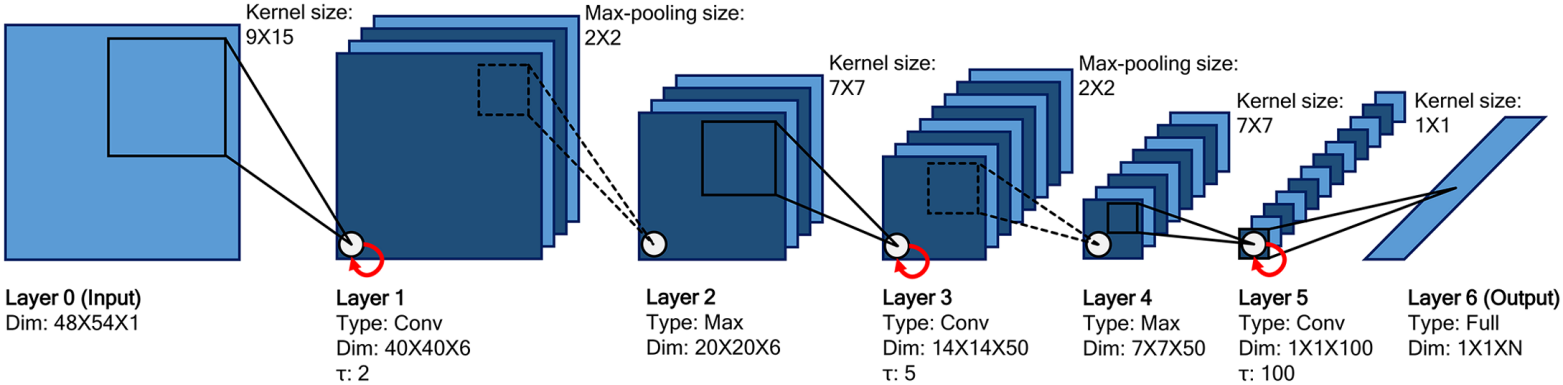
Architecture of the MSTNN. The architecture consists of one input layer, three convolutional layers, two max-pooling layers, and one fully-connected output layer. Convolutional layers apply convolution operations with kernels to previous layers (black solid lines). Max-pooling layers select maximum values within local windows from previous convolutional layers (black dotted lines). Each layer has a set of parameters: dimensions of layer (feature map column size×feature map row size×number of feature maps), kernel size, and max-pooling size. Only the convolutional layers have an additional time constant parameter *τ* (red solid arrow), which plays a key role in this model. The higher convolutional layer has a larger time constant than the lower convolutional layer. Layer 6 is the softmax activation function used for classification (*N* is the number of classes).

The network consists of 7 layers: one input layer (layer 0), three convolutional layers (layers 1, 3, and 5), two max-pooling layers (layers 2 and 4), and one fully-connected layer (layer 6) (see Fig 1). Layer 0 is the input layer, which has only one feature map size of 48×54, and contains the raw input image. Layer 1 is a convolutional layer that has 6 feature maps of size 40×40. Layer 1 convolutes layer 0 with a kernel size of 9×15. These feature maps are encoded via dynamic activities of (40×40×6) leaky integrator neural units with their time constant *τ* set to 2.0. Layer 2 is a max-pooling layer that has 6 feature maps of size 20×20. The number of feature maps of the max-pooling layer is the same as that of its previous convolutional layer because each feature map of the max-pooling layer and convolutional layer are coupled. Each feature map in layer 2 takes a maximum value within local patches with a size of 2×2 from the corresponding feature map in layer 1. Layer 3 is a convolutional layer encompassing 50 feature maps of size 14×14 and a time constant *τ* set to 5.0. Layer 3 convolutes layer 2 with a kernel size of 7×7. Layer 4 is a max-pooling layer that has 50 feature maps of size 7×7. Each feature map in layer 4 takes a maximum value within local patches with a size of 2×2 from the corresponding feature map in layer 3. Layer 5 is a convolutional layer that has 100 feature maps of size 1×1 and a time constant *τ* set to 100.0. Layer 5 convolutes layer 4 with a kernel size of 7×7. Layer 6 is a fully-connected layer which generates the categorical outputs encoded by a set of static neural units using the softmax activation function. The number of neurons in layer 6 is the same as the number of classes in a dataset. Each neuron in layer 6 is fully-connected with all the neurons of the 100 feature maps in layer 5.

## Results

### Weizmann Dataset and Data Preprocessing

**Fig 2:**
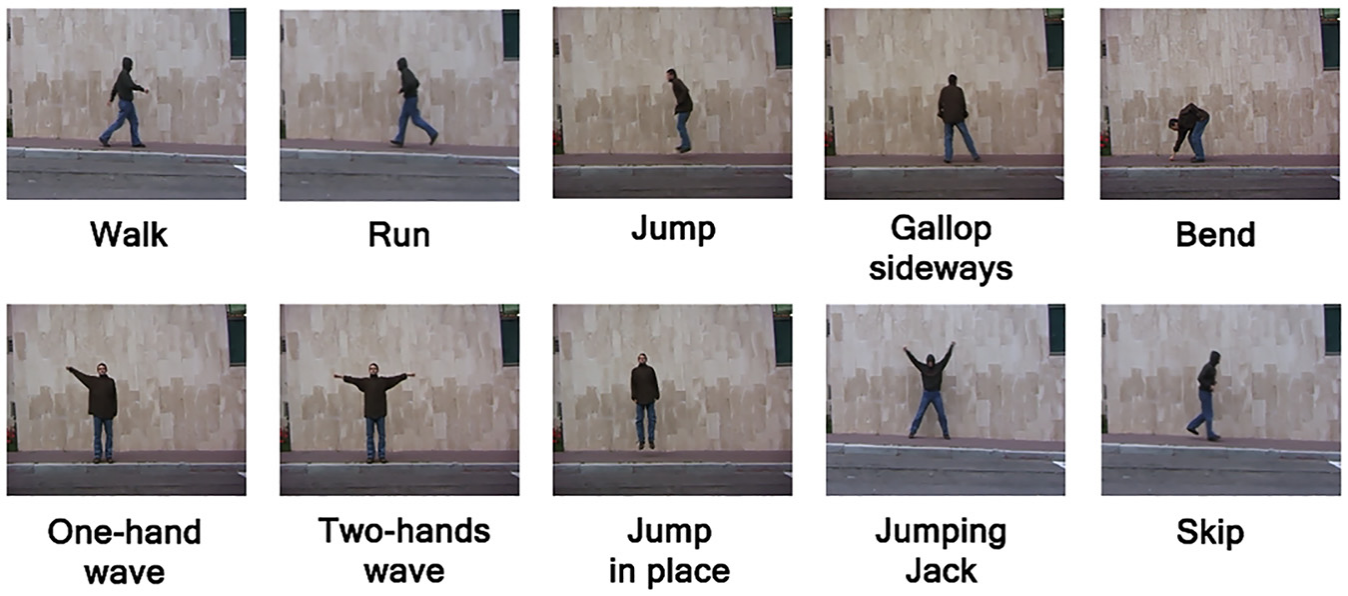
A sample frame for each action from the Weizmann dataset.

The Weizmann dataset [19] is provided by the Weizmann Institute of Science for single human action recognition. It contains 90 low resolution (180×144) visual sequences showing 10 human actions (walk, run, jump, gallop sideways, bend, one-hand wave, two-hands wave, jump in place, jumping jack, and skip), each performed by nine subjects (see Fig 2). Each visual sequence is about three seconds long. The view point and background are static.

In all experiments, we used aligned foreground silhouettes by background subtraction using background sequences and alignment based on subject position which were already included in the Weizmann dataset. We normalized all silhouette frames into the same size of 48×54.

### Resulting Recognition

The recognition by the two baseline models (the CNN and 3D CNN) was done using general majority voting, which encompasses recognition results over the entire time span of the input. In the case of the MSTNN, the visual sequences were recognized by majority voting of the recognition results generated upon the introduction of the black frames following the delay response method.

### Evaluation Protocol

For the evaluation protocol, we used leave-one-subject-out cross-validation. With nine subjects in the Weizmann dataset, eight subjects were selected for training, and the remaining subject was selected for testing. During the evaluation process, the network was trained for 50 epochs. To verify generalization to a testing subject, the average of nine trials with different testing subjects was calculated first, and then, this procedure was repeated with different random initialization seeds. The recognition rate was averaged over five different seeds and the highest recognition rate among 50 epochs was reported. The recognition performance was rounded off to the first decimal place.

### Prototypical Action Recognition on the Weizmann Dataset

The first experiment evaluated the basic performance of the MSTNN in recognition-by-learning for 10 relatively simple prototypical human actions from the Weizmann dataset. The MSTNN achieved 95.3% accuracy in the tests for recognition, comparable to the two baseline models: the CNN (92.9%) and 3D CNN (96.2%). See detail parameter settings for the two baseline models in S1 and S2 Tables. Schindler and Van Gool [23] showed that simple human actions can be sufficiently recognized using very short snippets (1–7 frames). The experimental results for prototypical action recognition were also consistent with their finding. Despite considering the time domain, the recognition accuracies of the MSTNN and baseline models were almost same.

**Fig 3:**
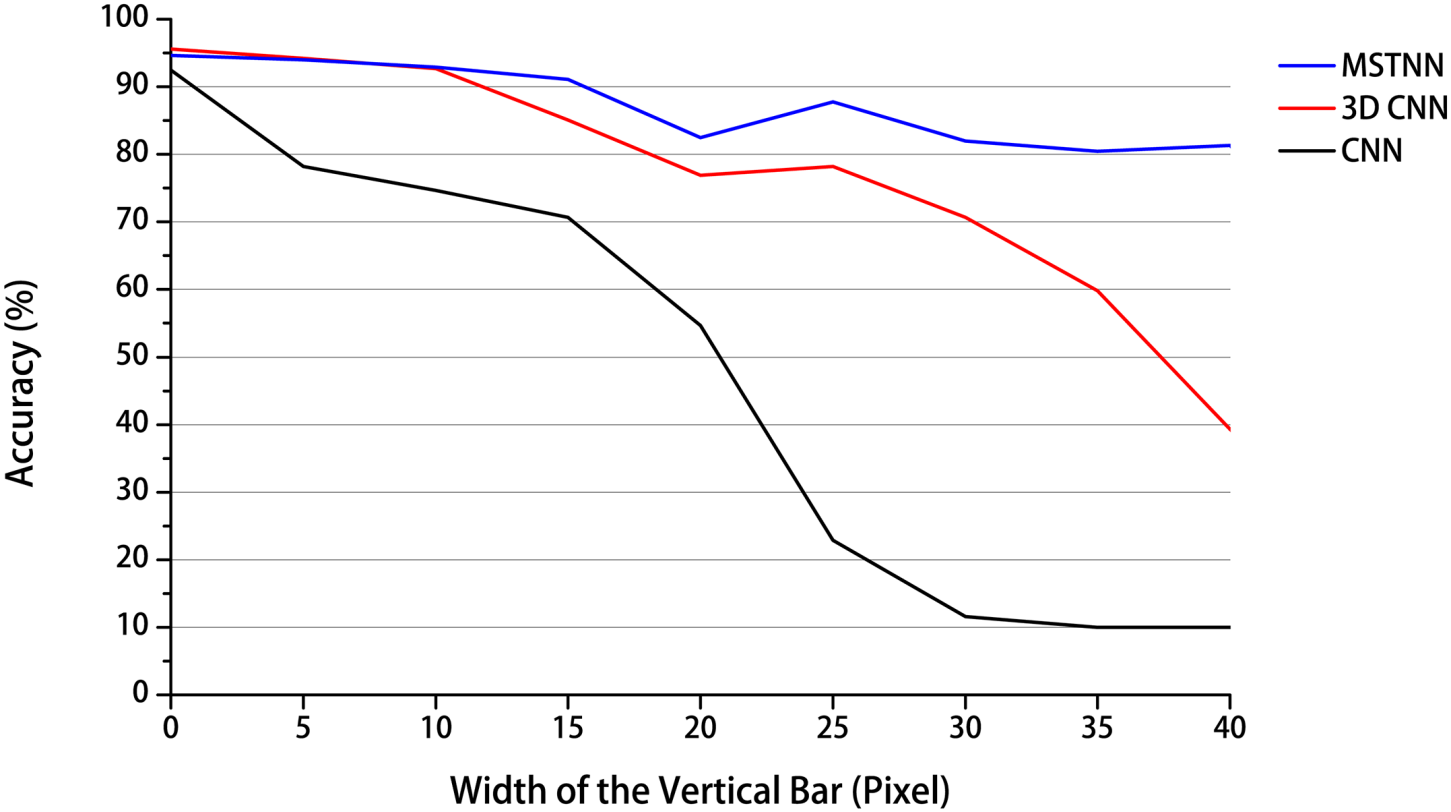
Recognition accuracy with respect to different degrees of occlusion. The vertical axis represents the recognition accuracy obtained as described in the text and the horizontal axis represents the width of the vertical bar, which corresponds to the degree of occlusion. Originally, the recognition accuracies of the MSTNN and two baseline models were similar (width of the vertical bar = 0). However, the recognition accuracy of the MSTNN was vastly superior to those of the two baseline models when the width of the vertical bar increased from 5 to 40 pixels stepping by 5 pixels.

Next, we conducted an occlusion experiment within the framework of the prototypical action recognition experiment to examine the effect of dynamic occlusion on the recognition performance in all three models. For the occlusion experiment, an original prototypical action video was artificially occluded by black vertical stripes moving horizontally. The interval between the vertical bars of the stripes was set to 5 pixels, and the stripes moved by 2 pixels per frame from right to left. The recognition accuracy was calculated according to the evaluation protocol, except for the selection of the highest accuracy among the epochs, because we used the network models previously trained in the last experiment for the current experiment. The experimental results show that the recognition accuracies of all three models started to decrease from the original ones without occlusion while increasing the width of the vertical bar from 5 to 40 pixels stepping by 5 pixels shown in Fig 3. However, there were differences in the performances among the three models. In the case of the CNN using only spatial information at each frame, the recognition accuracy rapidly deceased to the chance accuracy rate (10%), especially after the width of the vertical bar became wider than 15 pixels. However, the other two models, especially the MSTNN using both spatial and temporal information, were much robust for occlusion than the CNN in the recognition of dynamically occluded visual patterns. This is because the temporal information in the MSTNN and 3D CNN could compensate the spatial information lost by the occlusion. Especially in the case of the MSTNN which showed the highest performance among the three models, it is believed that the occlusion was not fatal for recognition because the spatial information temporarily occluded by the stripes can be preserved in the dynamic neural units (or leaky integrator neural units) with larger time constants.

### Recognition of Sequentially Combined Prototypical Actions

In the second experiment, the MSTNN was tested with visual sequences containing sequential combinations of prototypical human action patterns for the purpose of examining its capability for the contextual recognition of dynamic visual image patterns. A total of 81 visual sequences from the nine concatenated action categories were synthesized by concatenating two prototypical actions out of three (jump in place (JP), one-hand wave (OH), and two-hands wave (TH)) in the Weizmann dataset for all possible combinations done by the nine subjects. The number of frames per prototypical action used for the synthesis was 42 frames by cutting 1–42 frames from the original videos. The total number of frames for each concatenated action was 99 frames including 15 black frames. The resulting accuracy of recognition was 81.5%. See S1 Video for the contextual recognition processes in this task. Comparative studies with the CNN and 3D CNN were not done in this experiment because neither employ a temporal processing mechanism adequate for the contextual recognition task at hand. For instance, the 3D CNN model can only maintain temporal information within the restricted temporal dimension of the cube formed by stacking multiple contiguous frames together. Hence, in the second experiment, the temporal dimension size of the cube given to the 3D CNN should be large enough to extract the contextual information. However, the 3D CNN is practically limited to a small number of contiguous frames because the computation complexity greatly increases with the temporal dimension size of the cube.

**Fig 4:**
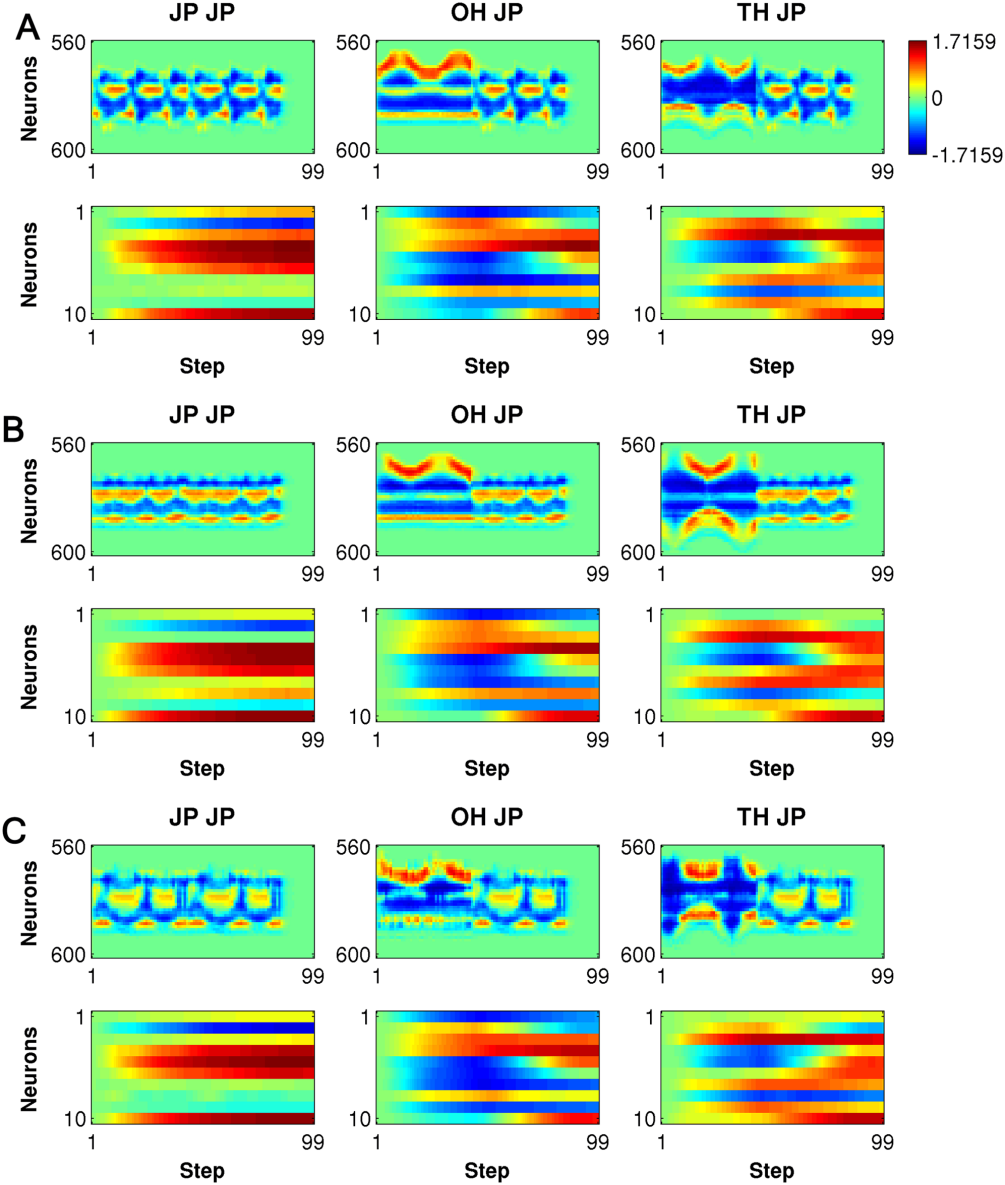
Time developments of the internal dynamics during the recognition of the concatenated actions. The neural activities are shown for those in layer 1 (*τ* = 2.0) and layer 5 (*τ* = 100.0). In each plot, the vertical axis represents the indices of the neurons and horizontal axis represents time steps. Specifically, the plots show the activities of 40 of 9600 encoded neurons in layer 1 (first row) and the activities of 10 of 100 neurons in layer 5 (second row) during JP → JP (first column), OH → JP (second column), TH → JP (third column) demonstrated by three different subjects for (A), (B) and (C).

**Fig 5:**
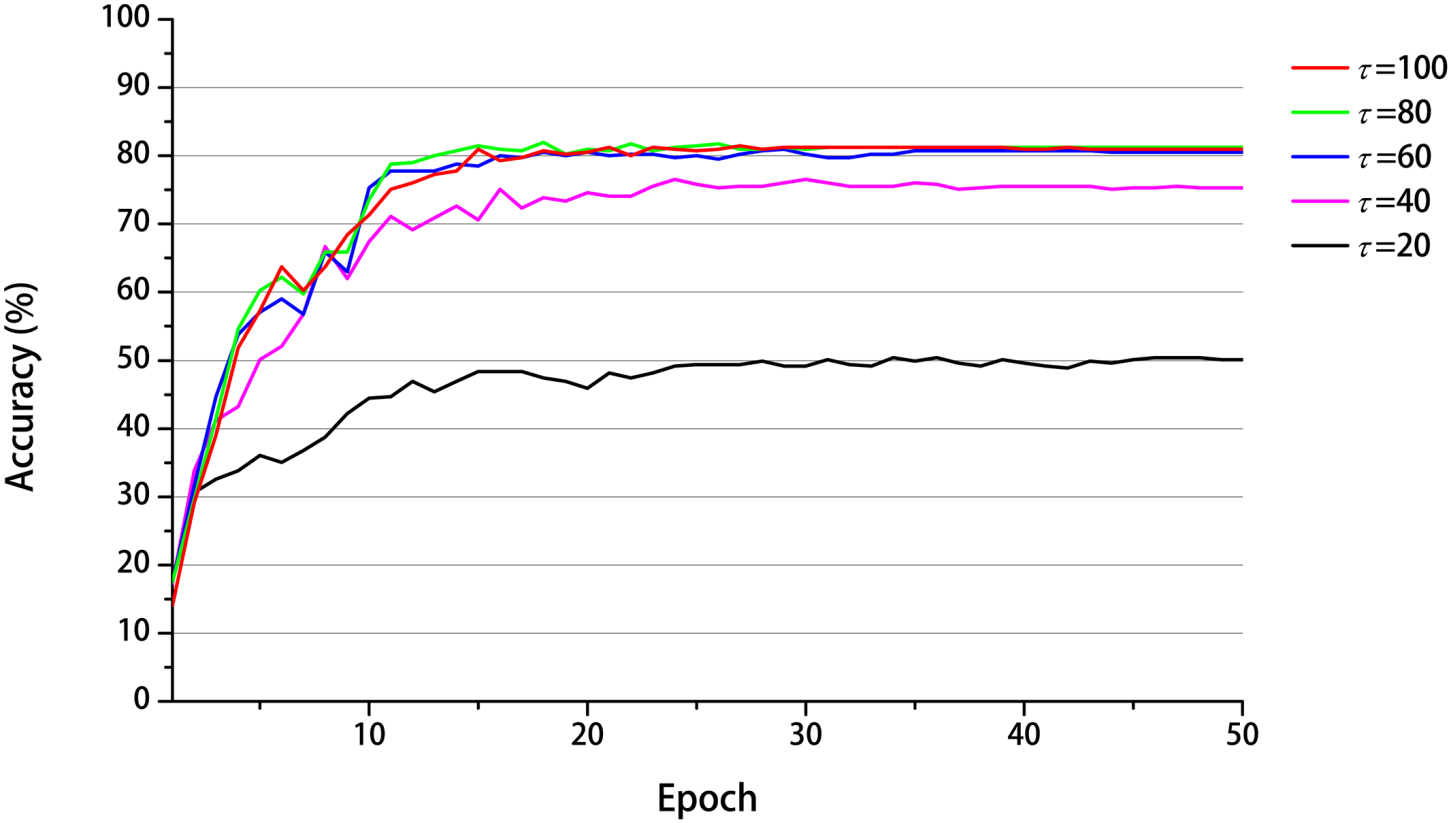
Development of the recognition accuracy with different time constants assigned for layer 5. The vertical axis represents the recognition accuracy obtained from the evaluation protocol and the horizontal axis represents epochs during the training phase. By changing the time constant from large (*τ* = 100.0) to small (*τ* = 20.0) in increments of 20, the accuracy decreased dramatically.

The task of recognizing concatenated actions is not trivial because the network has to preserve the memory of the first action when it generates the categorical outputs for all the concatenated actions at the end in the delay response method. To clarify the underlying mechanism, we conducted an analysis of the internal dynamics. In the MSTNN, layers 1, 3, and 5 correspond to the fast, middle, and slow layers, respectively. Fig 4 shows the time developments of the activities of 40 representative neural units in layer 1, with a smaller time constant (*τ* = 2.0), and the activities of 10 representative neural units in layer 5 with a larger time constant (*τ* = 100.0), for three different concatenated actions (JP → JP, OH → JP, and TH → JP) demonstrated by three different subjects. The neural activities in the fast layer showed detailed rhythmic patterns corresponding to the repetitive nature of the actions such as jumping in place or waving hands repeatedly. Meanwhile, the neural activities in the slow layer expressed steady patterns during the same movements, dramatically adopting other steady patterns when the actions changed. More importantly, the among-subjects-variances of the slow layer activities were smaller than those of the fast layer activities for the concatenated actions, showing an attenuation or “attunement” to the exhibited features. Furthermore, when the same action → such as “jump in place” → was preceded by different actions, the slow layer activities also developed characteristic differences. Briefly, the slow layer retains a memory of the first action, self-organizing such that this history is represented in the activation patterns during and after the perception of the second action. These results show how inter-subject contextual recognition could be achieved, with the slower neural activities in layer 5 which is essential to the success of the MSTNN. Thus, we examined how changes in the timescale constraint imposed on the network could affect the success of the contextual recognition. The recognition performance of the model was tested by changing the time constant in layer 5 from 100 to 20, in increments of 20, with the time constants at the lower layers fixed at the original values. The recognition accuracy deteriorated as the time constant was decreased shown in Fig 5. The recognition accuracies from time constant 100 to 60 were almost the same because time constants over 60 proved to be enough to memorize the first action category.

**Fig 6:**
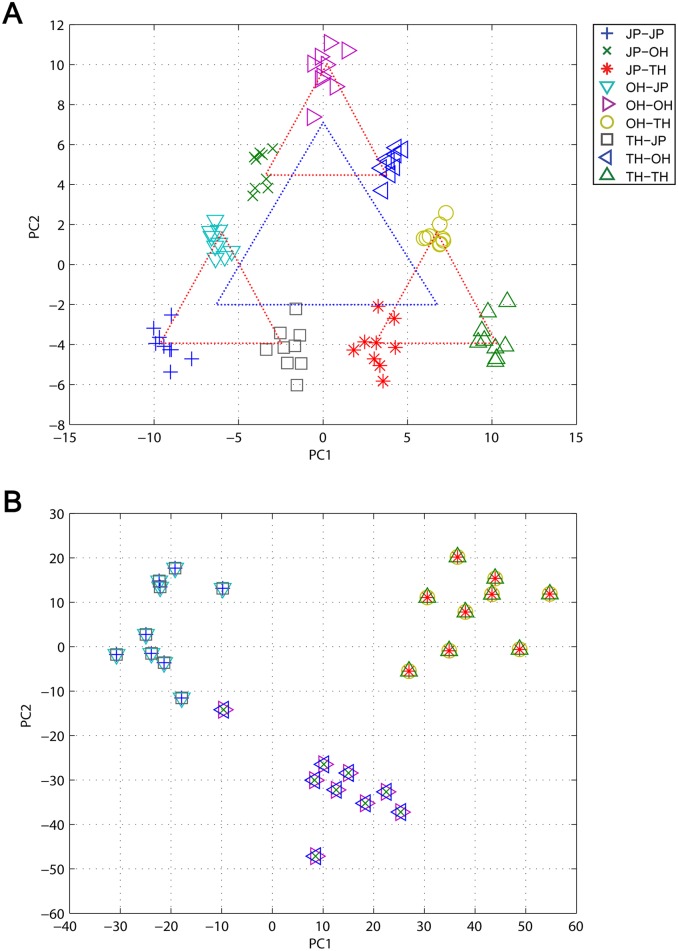
Snapshot of the internal dynamics of (A) layer 5 and (B) layer 3 plotted on the two dimensional PCA space. (A) PCA results on the internal dynamics at the end of all the concatenated actions (time step = 99) in layer 5. Nine groups corresponding to the nine different concatenated action categories appeared in the plot. Those nine groups presented themselves as three large clusters arranged according to the second action categories (blue dotted triangle), and each large cluster was also sub-divided into three isomorphic sub-clusters according to the first action categories (red dotted triangle). (B) PCA results on the internal dynamics at the end of the second action of all the concatenated actions (time step = 84) in layer 3. Three clusters were organized based on the current action category.

The functional hierarchy developed in the network was examined with principal component analysis (PCA) results to measure the activation states in the slow layer at the end of all the concatenated actions. When plotted, nine groups corresponding to the nine concatenated action categories emerged, with the groups arranged according to the first and last actions. Three large clusters (dependent on the second action) and three sub-clusters (dependent on the first action) are represented by blue and red triangles, respectively, in Fig 6A. Regardless of the scale, the bottom left, top center, and bottom right vertices of each triangle correspond with JP, OH, and TH, respectively. Interestingly, with the same PCA for the activation states in the middle layer at the end of the second action of all the concatenated actions, this type of self-similar structure across different scales was not observed in Fig 6B. Only three clusters appeared in the plot grouped merely on the current action category, regardless of the previous action.

Importantly, these results suggest how a functional hierarchy can be articulated for the recognition of visual patterns to be used in the decompositional analysis of action sequences. In the proposed model, the fast layer performs feature analysis for spatio-temporal patterns at the pixel level, and the middle layer affects the categorical representation of currently perceived actions using that feature information. Finally, the slow layer represents combinations of action sequences by developing self-similar structures between two different cluster scales. This self-similar structure is generated by applying the same dynamic function in the slow layer recursively with compositional action category inputs from the middle layer. This can be inferred from the nonlinear dynamic system theory of iterated function systems (IFS) [24, 25].

## Discussion

Although the capabilities of spatial and temporal processing in the CNN and leaky integrator models have been extensively explored, the current study represents the first time that these two principles have been combined into one single model, the MSTNN. Moreover, to our knowledge, this is the first demonstration of contextual recognition of pixel level dynamic visual image patterns based solely on self-organization through learning of exemplars. The experimental results show that a spatio-temporal hierarchy was successfully developed by applying both spatial and temporal multi-scales constraints on local neural activity in the neural network model. The evaluation experiments using the Weizmann dataset showed that (1) the hierarchical spatio-temporal processing mechanism achieved robust categorization performance by compensating spatial information when visual patterns of the prototypical actions were temporarily occluded and (2) the model also achieved contextual recognition of concatenated prototypical action patterns which cannot be attained by conventional models such as the CNN and 3D CNN models. In short, the main contribution of this work is to show that equipping a neural network architecture with a hierarchical structure, in both space and time, enables it to recognize moving patterns and sequences of visual movements. Using supervised learning, we effectively show that a necessary architectural feature of this recognition ability rests on a gradient of temporal scales with slower dynamics deeper or higher in the hierarchy. This confers a robustness that enables feed-forward architectures to recognize movement patterns, even in the presence of occlusion, and indeed, recognize sequences of movements.

Because humans as well as other animals use vision as a primary sensory channel for attaining information from a dynamically changing world, their visual cortex should deal with information processing not only for the spatial domain but also for the temporal domain simultaneously. As mentioned briefly in the introduction, spatial information processing in the visual cortex has been studied intensively [1–4, 17]. Multiple spatial scales hierarchy has been clearly observed in the visual cortex, especially the ventral pathway known to handle object recognition [26, 27]. The ventral pathway is organized with spatial hierarchy by means of developing spatial features at each local receptive field. More specifically, the primary visual cortex (V1) extracts relatively simple features, such as edges, bars, and gratings, from smaller spatial receptive fields, and such extracted simple features are combined into more complex features in higher levels (V2 and V4) having larger spatial receptive fields. Finally in the inferior temporal area TE, complex object features are formed by integrating information over large receptive fields.

However, how the visual cortex successfully extracts temporal information latent in dynamic visual image patterns is not well understood yet. We hypothesized that temporal information processing in the visual cortex might use the multiple scales hierarchy like the multiple spatial scales hierarchy assumed in the ventral pathway of the visual cortex. Hasson et al. [18] showed that short time scales of processing (4±1s) were observed in the early visual areas (V1 and the middle temporal (MT) area), while long timescales of processing (36±4s) were observed for high-order areas (the posterior lateral sulcus (LS), temporal parietal junction (TPJ), and the frontal eye field (FEF)). In addition, studies on other brain areas related to motor control support the idea of multiple timescales hierarchy. First, as shown by Tanji and Shima [28], there is a timescale difference in the buildup of neural activation dynamics between the supplementary motor area (with slower dynamics spanning timescales on the order of seconds) and M1 (with faster dynamics on the order of a fraction of a second) immediately before action generation. Considering this, Kiebel et al. [29], Badre and D→Esposito [30], and Uddén and Bahlmann [31] proposed a similar idea to explain the rostral-caudal gradient of timescale differences by assuming slower dynamics at the rostral side (the prefrontal cortex (PFC)) and faster dynamics at the caudal side (M1) in the frontal cortex to account for a possible functional hierarchy in the region.

Based on multiple spatial scales hierarchy based on prior studies that were mainly conducted for the ventral visual pathway and multiple timescales hierarchy that we assumed also for the visual cortex, this paper proposes that (1) the visual cortex could use the spatio-temporal hierarchy to extract abstract information from complex dynamic visual input in the retina and (2) the spatio-temporal hierarchy could be developed in the course of learning by imposing multiple scales constraints both in space and time simultaneously in local neural activity. Our model simulation experiments verified that the proposed principle works effectively. However, it is still a matter of debate whether such spatio-temporal hierarchy could be developed in either the ventral pathway or the dorsal one or whether there are dense interactions between these two. In fact, self-supervised learning of natural dynamic scenes statistics has been shown to produce a hierarchical dissociation of receptive field properties that bares a strong resemblance to the ventral and dorsal pathways (including magnocellular and parvocellular pathway differences and the functional segregation seen in the stripe structures of V2). These simulations again speak to the increasing size of the spatio-temporal receptive fields as one ascends the visual hierarchy [32].

When considering the basic principles in recognizing hierarchically organized spatial patterns, it is known that there are two different types of basic models. One is a non-predictive model, and the other is a predictive model. The distinction between the non-predictive and predictive models is based on the presence of recurrent or backward connections. The non-predictive model, such as the CNN [6], achieves visual categorization through a strictly feed-forward, or bottom-up, mechanism of details in the lower levels to abstractions in the higher levels. Because of its simple architecture, the non-prediction model is computationally cheap, and easily trained with a small number of epochs, while showing good recognition performance. However, the non-prediction model has inherently limited capability and is trained using a supervised learning approach that required labeled data for the target classification outputs. In contrast, the predictive model, such as a predictive coding model [33], attempts to solve the same problem using interactions between the top-down prediction and the bottom-up error regression, which can provide a more sophisticated computational mechanism compared to that of the non-predictive model. Additionally, the predictive model is trained by minimizing the prediction error between the target and its reconstruction without introducing labeled data. However, the crucial drawback of the predictive model if applied to the high-dimensional visual task domain is that it requires massive computational resources due to the prediction of high-dimensional data and its resultant iterative and interactive computations between the top-down and the bottom-up pathways.

For future study, it would be interesting to consider integration of both principles into one model. For example, it should be worthwhile to examine the possible advantages of adding the top-down prediction mechanism to the current MSTNN model to enhance its recognition capability both quantitatively and qualitatively.

## Supporting Information

S1 Video Demonstration video of the contextual recognition. The whole internal dynamics of the trained MSTNN are shown upon input of all nine different concatenated action categories performed by the test subject. The label on the bottom of the layer 0 indicates the current concatenated action category. Faster time scale dynamics of the lower layers were closely related to input dynamics. On the other hand, higher layers, especially layer 5, showed steady dynamics. Transition of steady dynamics in layer 5 was observed when a prototypical action was rapidly switched to another category. The MSTNN generated output results in the delay response method. By means of a large time constant, neurons in layer 5 can maintain their activation level to generate output following input completion as different from those in the lower layers.
(AVI)

S1 Table Parameter setting of the CNN. (DOCX)

S2 Table Parameter setting of the 3D CNN. (DOCX)
