# Supplementary material for: Self-Organization of Spatio-Temporal Hierarchy via Learning of Dynamic Visual Image Patterns on Action Sequences
Source: PLoS One. 2015 Jul 6;10(7):e0131214. doi: 10.1371/journal.pone.0131214 (PMC4492609; doi:10.1371/journal.pone.0131214)
Supplement: S2 Table — (DOCX) [file pone.0131214.s003.docx]

**S2 Table. Parameter setting of the 3D CNN**

|  | **Layer 0** | **Layer 1** | **Layer 2** | **Layer 3** | **Layer 4** | **Layer 5** | **Layer 6** |
| --- | --- | --- | --- | --- | --- | --- | --- |
| **Type** | Input | Conv | Max | Conv | Max | Conv | Full |
| **Feature map size** | 48 $\times$ 54 $\times$ 7 | 40 $\times$ 40 $\times$ 5 | 20 $\times$ 20 $\times$ 5 | 14 $\times$ 14 $\times$ 3 | 7 $\times$ 7 $\times$ 3 | 1 $\times$ 1 $\times$ 1 | 1 $\times$ 1 $\times$ 1 |
| **# of feature maps** | 1 | 6 | 6 | 50 | 50 | 100 | # of classes |
| **Kernel size** | - | 9 $\times$ 15 $\times$ 3 | - | 7 $\times$ 7 $\times$ 3 | - | 7 $\times$ 7 $\times$ 3 | 1 $\times$ 1 $\times$ 1 |
| **Pooling size** | - | - | 2 $\times$ 2 | - | 2 $\times$ 2 | - | - |
